# Smoking Increases the Risk of Hepatocellular Carcinoma and Cardiovascular Disease in Patients with Metabolic-Associated Fatty Liver Disease

**DOI:** 10.3390/jcm12093336

**Published:** 2023-05-08

**Authors:** Jeong-Ju Yoo, Man Young Park, Eun Ju Cho, Su Jong Yu, Sang Gyune Kim, Yoon Jun Kim, Young Seok Kim, Jung-Hwan Yoon

**Affiliations:** 1 Division of Gastroenterology and Hepatology, Department of Internal Medicine, Soonchunhyang University Bucheon Hospital, Gyeonggi-do 14584, Republic of Korea; puby17@naver.com (J.-J.Y.); mcnulty@schmc.ac.kr (S.G.K.); liverkys@schmc.ac.kr (Y.S.K.); 2 Korea Institute of Oriental Medicine, Daejeon 34054, Republic of Korea; pmy10042@gmail.com; 3 Department of Internal Medicine and Liver Research Institute, Seoul National University College of Medicine, Seoul 03080, Republic of Korea; ydoctor2@snu.ac.kr (S.J.Y.); yoonjun@snu.ac.kr (Y.J.K.); yoonjh@snu.ac.kr (J.-H.Y.)

**Keywords:** smoking, hepatocellular carcinoma, cardiovascular disease, fatty liver

## Abstract

The association of smoking with hepatocellular carcinoma (HCC) or cardiovascular disease (CVD) has been reported, but the study of its relationship with metabolic-associated fatty liver disease (MAFLD) is limited. We aimed to investigate the effect of smoking on the incidence of HCC or CVD in MAFLD patients. Using the Korean nationwide health screening database, we analyzed subjects between 2001 and 2015. A total of 283,088 subjects including 110,863 MAFLD patients and 172,225 controls were analyzed. Smoking status was divided by non-smoker, ex-smoker, or current smoker. In the follow-up period, a total of 2903 (1.0%) subjects developed HCC, and the MAFLD group (1723, 1.6%) had a significantly higher incidence than the control group (1180, 0.7%). In the MAFLD group, current smokers showed significantly higher risk of HCC compared to non-smokers (adjusted HR 1.24, 95% CI 1.08–1.41), whereas the control group did not (adjusted HR 1.07, 95% CI 0.89–1.30). A total of 18,984 (6.7%) patients developed CVD, and the incidence was significantly higher in the MAFLD group (8688, 7.8%) than in the control group (10,296, 6.0%), similar to HCC. The risk of CVD in current smokers increased by 22% compared to non-smokers in the MAFLD group (adjusted HR 1.22, 95% CI 1.15–1.30) and by 21% (adjusted HR 1.21, 95% CI 1.13–1.29) in the control group. Based on sex stratification, men showed increased incidence of both HCC and CVD by smoking, whereas women had only increased risk of CVD. Smoking significantly increases the incidence of HCC and CVD in MAFLD patients; thus, it is highly recommended to quit smoking completely in the population with MAFLD.

## 1. Introduction

Recently, the concept of “metabolic (dysfunction)-associated fatty liver disease (MAFLD)” has emerged in studies of fatty liver disease [1]. Unlike nonalcoholic fatty liver disease (NAFLD), which excludes concomitant liver diseases and/or heavy alcohol consumption, MAFLD focuses on the role of metabolic dysfunction and does not exclude other etiologies of liver diseases (e.g., alcohol). Several studies have confirmed that the renaming/definition change from NAFLD to MAFLD is useful for identifying metabolically complex liver diseases and identifying adults with high risk of adverse outcome [2,3]. MAFLD elevates the risk of cardiovascular disease (CVD), stroke, and chronic kidney disease [4]. Moreover, it significantly increases the risk of fibrosis, liver cirrhosis, and even hepatocellular carcinoma (HCC) [3]. Globally, the proportion of fatty liver disease has increased, whereas the prevalence of viral hepatitis has decreased [5]. In this era of metabolic disease, non-viral risk factors (e.g., smoking) have grown in importance for management of liver disease [6,7].

Smoking is the leading cause of preventable disease and death in the United States, causing approximately 480,000 deaths annually, accounting for one in five deaths due to tobacco use [8]. The adverse effects of smoking on lungs and the cardiovascular system are well studied. To date, smoking was shown to promote CVD in patients with steatohepatitis and in liver transplant recipients [9,10,11]. In addition, smoking has been known to increase the incidence of de novo or recurrent HCC in chronic viral hepatitis patients [12,13,14]. However, the detrimental effects of smoking on the liver have not been studied well, especially in relation to fatty liver disease. Therefore, this study aims to investigate the effect of smoking on the incidence of HCC and CVD in patients with fatty liver disease, especially MAFLD.

## 2. Materials and Methods

### 2.1. Data Source and Study Population

We used the database of the Korean National Health Insurance Service (NHIS)-National Sample Cohort, which represent approximately 2% of the entire Korean population. The database contains de-identified data including demographic and claimed information based on International Classification of Diseases, 10th revision (ICD-10). The NHIS medical check-up database specifically provides the results of biennial health examinations and the data of lifestyle and behavior. A detailed description of the cohort has been reported previously [15].

A total of 514,866 adults aged 20 years or older who underwent health screening examinations between 2002 and 2015 were included. Subjects with missing information in the tobacco-related questionnaire or subjects who died in the first year of the follow-up period (n = 74,041) among the patients diagnosed with cardiovascular outcomes or HCC before index year (n = 31,369) were excluded, and we ascertained outcome events after a two-year lag (n = 19,632). Participants with a history of chronic viral hepatitis (B18), alcoholic liver disease (K70), primary biliary cholangitis (K74.3), autoimmune hepatitis (K75.4), Wilson’s disease (E83.0), and Budd-Chiari syndrome (I82.0) before the index date were further excluded from the control group (n = 52,000).

The Institutional Review Board of Seoul National University Hospital approved the current study (IRB No. E-1910-003-1066). Informed consent was waived from the IRB, as only de-identified information was used.

### 2.2. Data Collection

NHIS provides information on smoking to subjects based on data collected through questionnaires. The questionnaire surveys items related to smoking status, smoking period, and daily smoking amount. Among these, subjects must select either (i) non-smoker, (ii) ex-smoker, and (iii) current smoking for “smoking status”. The definition of a non-smoker is “a person who has never smoked”, an ex-smoker is “a person who smoked in the past but has now stopped”, and a current smoker is “a person who still smokes”. Lifestyle variables obtained through self-administered questionnaires included the following information: alcohol consumption (none, mild (less than 140 g/week for men, 70 g/week for women), moderate (140 g/week ≤ for men < 210 g, 70 g/week ≤ for women < 140 g), or heavy alcoholics (more than 210 g/week for men, 140 g/week for women)), and regular exercise (more than 20 to 30 min of moderate-to-vigorous activity at least three times per week). Comorbidities were defined using ICD-10 diagnostic codes, prescription information before the health examination, and blood test results. Liver cirrhosis was defined by the ICD-10 codes K702, K703, K74, K766, or K767.

### 2.3. Definition of MAFLD and Control Group

Because ultrasonography is not included in the NHIS health examination, fatty liver index (FLI), the alternative to imaging modalities in large epidemiologic studies, was used for assessment of steatosis [16]. FLI scores range from 0–100, with <30 representing low risk for fatty liver and ≥60 representing high risk [17]. The lower cutoff of FLI ≥ 30 was used in this study [18,19]. The hepatic steatosis index (HSI) was also calculated for the purpose of sensitivity analysis to validate the FLI results. The equations of FLI and HIS are as follows:

Fatty liver index = 1/(1 + exp(–x*)) × 100

x* = 0.953 × log_e_ (triglycerides) + 0.139 × BMI + 0.718 × log_e_ (γ-glutamyl-transferase) + 0.053 × (Waist circumference) − 15.745

Hepatic steatosis index = 8 × ALT/AST ratio + BMI (+2 if diabetes; +2 if female)

Among patients with FLI scores of 30 or higher, MAFLD group was defined by those having one or more of the following criteria: (a) overweight or obese (body mass index (BMI) ≥ 23 kg/m^2^), (b) diabetes, and (c) at least two or more of the following metabolic abnormalities: (i) abdominal circumference of 90 cm or more for men and 80 cm or more for women, (ii) blood pressure higher than 130/85 mmHg or taking antihypertensive medications, (iii) serum triglyceride higher than 150 mg/dL or taking lipid-lowering drugs, (iv) high-density lipoprotein cholesterol (HDL) less than 40 mg/dL in men and less than 50 mg/dL in women, and (v) fasting blood glucose above 100 mg/dL [1]. As C-reactive protein levels and homeostasis model assessment of insulin resistance scores were not included in the NHIS screening program, these criteria for metabolic abnormalities were not used in this study.

The control group was defined as subjects without underlying liver disease such as chronic viral hepatitis (B18), alcoholic liver disease (K70), cirrhosis (K74), primary biliary cholangitis (K74.3), autoimmune hepatitis (K75.4), Wilson’s disease (E83.0), and Budd-Chiari syndrome (I82.0) among those with FLI scores of less than 30. Finally, a total of 283,088 subjects including 110,863 MAFLD patients and 172,225 controls were analyzed.

### 2.4. Study Outcome

The study outcomes were the occurrence of HCC or CVD. HCC was defined by ICD-10 code C22.0 with the registration of special cancer claim code V193. CVD was defined as myocardial infarction or stroke. Myocardial infarction was defined as the recording of ICD-10 codes I21 or I22 during hospitalization or these codes having been recorded at least two times. Stroke was based on the ICD-10 codes I63 or I64 during hospitalization or having two or more medical records. The study population was followed from initial to the date of study outcomes, censoring date, or until 31 December 2015, whichever came first.

### 2.5. Statistical Analyses

Continuous variables are presented as means ± standard deviations (SDs) and as proportions for categorical variables, unless otherwise indicated. The Student’s *t*-test for continuous variables and χ^2^ test for categorical variables were used to analyze differences between the groups. Cox proportional hazard models adjusted for age, sex, smoking status, alcohol consumption, regular exercise, body mass index (BMI), diabetes, hypertension, dyslipidemia, and presence of cirrhosis were performed.

Statistical analyses were performed using SAS version 9.4 (SAS Institute, Cary, NC, USA) and R version 3.2.3 (The R Foundation for Statistical Computing, Vienna, Austria, http://www.Rproject.org accessed on 12 December 2022). A two-sided *p* value < 0.05 was considered statistically significant.

## 3. Results

### 3.1. Baseline Characteristics of the Study Population

Baseline characteristics of the MAFLD and control groups are shown in Table 1. Compared to the control, the MAFLD group showed higher BMI (25.8 ± 2.6 vs. 22.3 ± 2.2 kg/m^2^) and was more likely to have diabetes (12.6% vs. 6.5%), hypertension (31.9% vs. 18.2%), and dyslipidemia (7.2% vs. 4.8%) (*p* < 0.001 for all). The MAFLD group had higher rates of current (26.7% vs. 14.0%) or ex-smokers (26.2% vs. 14.2%) than the control group, and the proportion of moderate-to-heavy drinkers was higher in the MAFLD group (33.4% vs. 11.1%). In the blood test results, the mean values of cholesterol of total and LDL, triglyceride, FLI, and hepatic steatosis index were higher in the MAFLD group than the control group (*p* < 0.001 for all, Table 1).

### 3.2. Incidence of HCC and CVD

Table 2 summarizes the outcome events of HCC and CVD. The definition of “time to event” was the time from diagnosis of MAFLD or health screening to onset of HCC or CVD. During the mean follow-up duration of 1813.1 ± 124.9 days, a total of 2903 (1.0%) patients developed de novo HCC. The MAFLD group (1723, 1.6%) had a significantly higher incidence of HCC than the control (1180, 0.7%). In terms of CVD, a total of 18,984 (6.7%) patients developed the disease, and similar to HCC, it was significantly higher in the MAFLD group (8688, 7.8%) than the control (10,296, 6.0%).

### 3.3. Association between Smoking and Incident HCC

**Figure 1:**
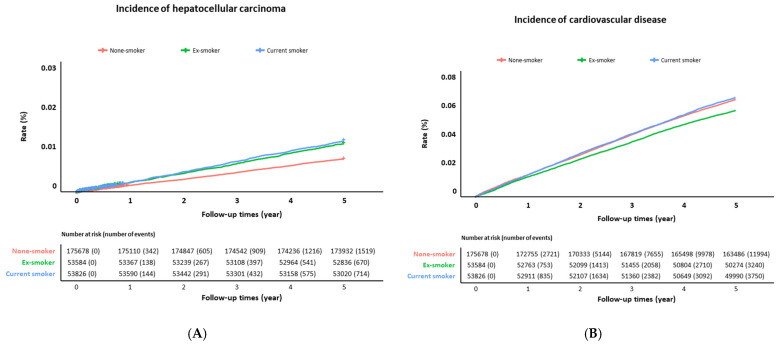
Incidence of (**A**) hepatocellular carcinoma and (**B**) cardiovascular disease according to smoking status.

Table 3 and Figure 1A show the incidence and risk of HCC depending on the smoking status. Compared to non-smokers, the ex- and current smokers showed 11% and 21% increase in the HCC risk, respectively (adjusted hazard ratio (aHR) 1.11, 95% confidence interval (CI) 1.00–1.24; aHR 1.21, 95% CI 1.09–1.35).

When subjects were classified into the MAFLD and control groups, the current smoker group showed a significantly higher risk of HCC (aHR 1.24, 95% CI 1.08–1.41) in the MAFLD group, whereas it was attenuated in the control group (aHR 1.07, 95% CI 0.89–1.30). In a subgroup analysis of MAFLD patients with cirrhosis, HR was in the same direction in the current smokers but no longer reached statistical significance because of the small number of cases.

### 3.4. Smoking on the Development of CVD

Table 4 and Figure 1B show the incidence and risk of CVD depending on the smoking status. Ex-smokers did not show significant differences in the risk of CVD compared with non-smokers. However, current smokers showed a 24% increased risk compared to the control (aHR 1.24, 95% CI 1.19–1.30). CVD risk in the current smokers was not significantly different in the presence or absence of MAFLD (MAFLD: aHR 1.22, 95% CI 1.15–1.30; control: aHR 1.21, 95% CI 1.13–1.29, respectively). Notably, among MAFLD patients with cirrhosis, the association between current smoking and CVD was strengthened but did not reach statistical significance due to the small number of patients.

### 3.5. Effect of Smoking in Stratification by Sex

Next, stratified analysis by sex was conducted for the occurrence of HCC and CVD for the MAFLD group only (Table 5). Patients were classified into two groups: those who experienced smoking at least once (ex-smoker or current smoker) and those who never smoked. Smoking is shown to be correlated to the higher risk of HCC (aHR 1.14, 95% CI 1.02–1.28, *p* = 0.02) and CVD (aHR 1.08, 95% CI 1.03–1.14, *p* = 0.003), respectively, in all the subjects. These results have sex differences. In men, smoking significantly increased the incidence of both HCC (aHR 1.14, 95% CI 1.02–1.29, *p* = 0.03) and CVD (aHR 1.08, 95% CI 1.02–1.14, *p* = 0.009), whereas women only showed a significant relationship between CVD and smoking (aHR 1.34, 95% CI 1.11–1.61, *p* = 0.002) but not to a significant extent in HCC (aHR 1.23, 95% CI 0.72–2.12, *p* = 0.45).

## 4. Discussion

HCC has an incidence of 9.3 cases per 100,000 person-years worldwide, and the long-term mortality has not improved from 8.5 cases [20]. It accounts for about 22% of cancer-related deaths worldwide [21]. Recently, the proportion of HCC caused by fatty liver has increased, and it is predicted that by 2030, the incidence of fatty liver-related HCC will reach 122% in the United States and 117% in France [22]. Fatty-liver-associated HCC is known to be more dangerous because it can occur even in the absence of cirrhosis, which is a strong risk factor. Therefore, it is even more important to find preventable HCC risk factors in the fatty liver patient group. The current study focused more on smoking among the risk factors.

The effects of smoking on health can be roughly divided into three categories: (1) lung disease, (2) tumor, and (3) thrombosis or CVD [23,24,25]. In developed countries, smoking rates have gradually decreased by virtue of smoking cessation policies, but morbidity and mortality are still significantly higher in developing countries [26]. Historically, under the assumption that smoking is not likely to have a direct effect on the liver, there were limited studies showing that the liver as an organ is harmed by smoking. However, recent accumulated studies report that smoking affects the development of liver fibrosis and liver cancer. These studies include that smoking directly affects the liver through (1) toxic, (2) immunological, and (3) oncogenic mechanisms [8,27]. By mechanisms with toxic effects, smoking increases oxidative stress, which leads to liver fibrosis through stellate cell activation [28,29,30]. Immunologically, it induces lymphocyte apoptosis, activates CD8 cytotoxic T cells, and lowers the activities of CD4 T cells and NK cells [29,31,32]. The oncogenic mechanism of smoking is that substances such as 4-aminobiphenyl and vinyl chloride in cigarettes directly increase the risk of HCC [33,34] and inhibit p53, a tumor suppressor [30,35].

In viral hepatitis, smoking increases the incidence of HCC. A meta-analysis of 96 studies showed that smoking increased liver cancer by 51%, and ex-smokers especially presented 12% of the incline [36]. There have been studies showing that the normal population has an increased risk of HCC by smoking [37], but there were no reports about the relation of smoking to liver cancer, limited only to fatty liver disease. For the first time to our knowledge, the present study demonstrated that smoking increases the risk of HCC by approximately 24% in patients with MAFLD. This risk is lower than the smoking effect on HCC in previously known patients with viral hepatitis.

When it comes to fatty liver, smoking also increases its incidence in the normal population and is associated with a poor prognosis in patients with the same condition [38]. Current smoking was presented to be a strong risk factor for mortality or liver transplantation in patients with biopsy-confirmed fatty liver for 12.6 years follow-up (HR 2.6, 95% CI 1.67–4.1) [39].

While CVD was known to be one of the biggest causes of death [39], the current study demonstrated significantly higher CVD incidence in the MAFLD group of current smokers. The major risk factors for fatty-liver-associated HCC are diabetes and obesity, and its relative risk is increased by about 1.4 to 4.1 [40,41]. Our findings suggest that smoking should be added as a new risk factor, thus requiring strict smoking cessation in patients with MAFLD. However, there are only a few studies that demonstrate the synergistic effect of smoking on metabolic comorbidity and its controversy. In a study conducted in the United States, no synergistic effect between diabetes and smoking in the development of HCC [42] was reported, but other studies showed a synergism between obesity and smoking in the development of HCC [43]. Therefore, it is hard to conclude that smoking is particularly dangerous in patients with MAFLD compared to liver disease of other etiology.

A noteworthy part of our study is that ex-smokers have a reduced risk of HCC compared to current smokers. There were three large-scale meta-analyses on the relationship between smoking and HCC in patients with viral hepatitis or in a normal population [36,37,44]. All studies demonstrated that the risk of HCC in ex-smokers was significantly reduced compared to that of current smokers. In our study, subjects with MAFLD showed that the risk of liver cancer was reduced by about 24% to 4%, suggesting that strict smoking cessation is required. Even in patients who have already developed HCC, smoking increases the risk of recurrence and is associated with a poor prognosis. Therefore, it can be said that smoking cessation is the most important factor in all treatment stages of liver disease [14]. However, compared with the non-smoker group, the ex-smoker group had a lower risk of CVD in MAFLD. Our assumptions about the phenomenon are as follows: First, it is possible that smoking cessation itself lowered the risk of CVD while influencing other behavioral changes (e.g., exercise). In this case, not only smoking cessation but also complex risk factors such as weight management and exercise affect the CVD risk. In addition, we cannot exclude the sick-quitter effect because of the retrospective observational nature of our study. However, we tried to reduce this by excluding those who had a prior diagnosis of CVD or HCC before cohort entry, and we ascertained outcome events after a lag of two years. Second, in our study, we used a different definition of “ex-smoker” from the Centers for Disease Control and Prevention guidelines [45,46]. It is possible that this difference in classification influenced the results. Finally, even for ex-smokers, there is a difference in the risk of CVD between heavy and light smokers. Since the ex-smoker group is a relatively heterogenous group containing both heavy and light smokers, it is possible that this heterogenicity influenced the results.

Another finding in our study was that there were differences in risk between men and women. Cigarette smoking increased the incidence of both liver cancer and CVD in men but only increased the incidence of CVD in women. Differences in HCC between genders seem to be connected to both behavioral and biological elements. Wu et al. [47] reported that elderly women with HCC exhibit a higher prevalence of NAFLD/NASH and might be missed by existing monitoring recommendations. The causative processes responsible for these disparities have not been clearly identified; they may be biological, such as thrombin signaling, or associated with variations in smoking habits between males and females [48].

Our study has the following shortcomings: First, considering the period of occurrence in fatty-liver-associated HCC, the follow-up period is relatively short. Second, since we could not obtain the results of the dose response in smoking, it is hard to provide a criterion for exactly how much smoking is dangerous for the development of HCC or CVD. Third, our study was conducted only in Korea, a clustered population. Thus, there are limitations in applying it to the Western population, which is a relatively heterogenous group. Finally, risk factors for fatty-liver-associated HCC have not been established yet, so there might be other risk factors for HCC that we have not considered. For better insight, long-term studies are required on following topics: First, ex-smokers show the tendency to reduce the risk of HCC compared to current smokers, but research on how many years of smoking cessation is needed should be considered. Second, further study to determine how e-cigarettes affect the development of HCC is required.

In conclusion, smoking significantly increases the incidence of HCC and cardiovascular disease in patients with MAFLD, and therefore, it is strongly required that patients with this condition quit smoking.

## Figures and Tables

**Table 1:** Baseline characteristics of the study population.

Variable	Total	MAFLD	Control	*p*-Value *
	(n = 283,088)	(n = 110,863)	(n = 172,225)	
Age, years	58.6 ± 8.6	58.2 ± 8.2	58.8 ± 8.9	<0.001
Male	159,139 (56.2%)	83,636 (75.4%)	75,503 (43.8%)	<0.001
Body mass index, kg/m^2^	23.7 ± 2.9	25.8 ± 2.6	22.3 ± 2.2	<0.001
Waist circumference, cm	81.4 ± 8.4	88.1 ± 6.3	77.1 ± 6.5	<0.001
Systolic blood pressure, mmHg	125.1 ± 15.5	129.3 ± 15.0	122.4 ± 15.1	<0.001
Diastolic blood pressure, mmHg	77.6 ± 10.1	80.4 ± 9.9	75.8 ± 9.8	<0.001
Diabetes	25,109 (8.9%)	13,927 (12.6%)	11,182 (6.5%)	<0.001
Hypertension	66,676 (23.6%)	35,311 (31.9%)	31,365 (18.2%)	<0.001
Dyslipidemia	16,341 (5.8%)	8005 (7.2%)	8336 (4.8%)	<0.001
Smoking				<0.001
Non-smoker	175,678 (62.1%)	52,159 (47.0%)	123,519 (71.7%)	
Ex-smoker	53,584 (18.9%)	29,050 (26.2%)	24,534 (14.2%)	
Current smoker	53,826 (19.0%)	29,654 (26.7%)	24,172 (14.0%)	
Alcohol consumption				<0.001
None	155,868 (55.1%)	43,996 (39.7%)	111,872 (65.0%)	
Mild	70,870 (25.0%)	29,792 (26.9%)	41,078 (23.9%)	
Moderate	23,205 (8.2%)	13,995 (12.6%)	9210 (5.3%)	
Heavy drinker	33,145 (11.7%)	23,080 (20.8%)	10,065 (5.8%)	
Regular exercise	151,854 (53.6%)	61,999 (55.9%)	89,855 (52.2%)	<0.001
Total cholesterol, mg/dL	200.7 ± 37.0	205.4 ± 38.9	197.6 ± 35.4	<0.001
HDL cholesterol, mg/dL	55.0 ± 24.7	51.3 ± 26.3	57.4 ± 23.3	<0.001
LDL cholesterol, mg/dL	118.9 ± 36.7	116.9 ± 39.8	120.2 ± 34.5	<0.001
Triglyceride, mg/dL	139.4 ± 91.1	195.8 ± 111.1	103.2 ± 48.2	<0.001
Fatty liver index	28.8 ± 23.0	53.0 ± 17.2	13.2 ± 7.8	<0.001
Hepatic steatosis index	31.9 ± 4.3	34.9 ± 4.3	30.1 ± 3.2	<0.001
Follow-up period (days)	1104.1 ± 672.5	1111.6 ± 666.9	1093.2 ± 680.5	0.428

Data are expressed as mean ± standard deviation or number and percentage. * *p*-value was calculated between MAFLD and control group. Abbreviations: MAFLD, metabolic-associated fatty liver disease; HDL, high-density lipoprotein; LDL, low-density lipoprotein.

**Table 2:** Overview of study outcome.

	Total	MAFLD	Control	*p*-Value *
	(n = 283,088)	(n = 110,863)	(n = 172,225)	
**Outcome: hepatocellular carcinoma**				
Total follow-up (day)	1813.1 ± 124.9	1808.8 ± 143.3	1815.9 ± 111.4	<0.001
Event (n)	2903 (1.0%)	1723 (1.6%)	1180 (0.7%)	<0.001
Time to event (day)	1104.1 ± 672.5	1111.6 ± 666.9	1093.2 ± 680.5	0.43
**Outcome: cardiovascular disease**				
Total follow-up (day)	1758.3 ± 282.1	1747.4 ± 303.2	1765.4 ± 267.5	<0.001
Event (n)	18,984 (6.7%)	8688 (7.8%)	10,296 (6.0%)	<0.001
Time to event (day)	1063.1 ± 670.4	1053.2 ± 668.7	1071.3 ± 671.7	0.04

* *p*-value was calculated between MAFLD and control group. Abbreviations: MAFLD, metabolic-associated fatty liver disease.

**Table 3:** Effect of tobacco smoking on the development of hepatocellular carcinoma.

Group		Non-Smoker	Ex-Smoker	Current Smoker
Total	Total, no.	175,678	53,584	53,826
	Events, no.	1519	670	714
	Person-years	873,465	265,865	266,901
	Adjusted * HR	1 (ref)	1.11 (1.00–1.24)	1.21 (1.09–1.35)
MAFLD	Total, no.	52,159	29,050	29,654
	Events, no.	745	451	527
	Person-years	258,616	144,007	146,767
	Adjusted * HR	1 (ref)	1.04 (0.91–1.19)	1.24 (1.08–1.41)
Control	Total, no.	123,519	24,534	24,172
	Events, no.	774	219	187
	Person-years	614,849	121,858	120,134
	Adjusted * HR	1 (ref)	1.18 (0.99–1.42)	1.07 (0.89–1.30)
Subgroup: liver cirrhosis	Total, no.	166	126	156
	Events, no.	17	11	17
	Person-years	792	597	720
	Adjusted * HR	1 (ref)	0.89 (0.40–1.99)	1.25 (0.59–2.64)

Abbreviations: no., number; HR, hazard ratio. * Adjusted for age, sex, alcohol drinking, exercise, body mass index, diabetes, hypertension, dyslipidemia, and presence of cirrhosis.

**Table 4:** Effect of tobacco smoking on the development of cardiovascular disease.

Group		Non-Smoker	Ex-Smoker	Current Smoker
Total	Total, no.	175,678	53,584	53,826
	Events, no.	11,994	3240	3750
	Person-years	845,810	259,027	258,893
	Adjusted * HR	1 (ref)	0.95 (0.91–1.00)	1.24 (1.19–1.30)
MAFLD	Total, no.	52,159	29,050	29,654
	Events, no.	4583	1860	2245
	Person-years	248,422	140,148	142,167
	Adjusted * HR	1 (ref)	0.92 (0.86–0.97)	1.22 (1.15–1.30)
Control	Total, no.	123,519	24,534	24,172
	Events, no.	7411	1380	1505
	Person-years	597,388	118,880	116,726
	Adjusted * HR	1 (ref)	0.96 (0.90–1.03)	1.21 (1.13–1.29)
Subgroup: liver cirrhosis	Total, no.	166	126	156
	Events, no.	15	8	19
	Person-years	782	595	724
	Adjusted * HR	1 (ref)	0.84 (0.34–2.11)	1.79 (0.83–3.88)

Abbreviations: no., number; HR, hazard ratio. * Adjusted for age, sex, alcohol drinking, exercise, body mass index, diabetes, hypertension, dyslipidemia, and presence of cirrhosis.

**Table 5:** Effect of tobacco smoking stratified by sex.

			Total		Male		Female	
Outcome	Model	Smoking	Adjusted HR	*p*	Adjusted HR	*p*	Adjusted HR	*p*
HCC	Model 1 *	Smoking (−)	1 (ref)		1 (ref)		1 (ref)	
		Smoking (+)	1.17 (1.06–1.28)	0.001	1.05 (0.93–1.18)	0.43	1.09 (0.64–1.86)	0.75
	Model 2 ^†^	Smoking (−)	1 (ref)		1 (ref)		1 (ref)	
		Smoking (+)	1.12 (1.00–1.25)	0.056	1.14 (1.01–1.28)	0.03	1.10 (0.64–1.87)	0.74
	Model 3 ^§^	Smoking (−)	1 (ref)		1 (ref)		1 (ref)	
		Smoking (+)	1.14 (1.02–1.28)	**0.02**	1.14 (1.02–1.29)	**0.03**	1.23 (0.72–2.12)	0.45
CVD	Model 1 *	Smoking (−)	1 (ref)		1 (ref)		1 (ref)	
		Smoking (+)	0.79 (0.76–0.82)	<0.001	0.86 (0.82–0.91)	<0.001	1.28 (1.07–1.54)	0.008
	Model 2 ^†^	Smoking (−)	1 (ref)		1 (ref)		1 (ref)	
		Smoking (+)	1.06 (1.01–1.12)	0.03	1.05 (0.99–1.11)	0.08	1.32 (1.10–1.58)	0.003
	Model 3 ^§^	Smoking (−)	1 (ref)		1 (ref)		1 (ref)	
		Smoking (+)	1.08 (1.03–1.14)	**0.003**	1.08 (1.02–1.14)	**0.009**	1.34 (1.11–1.61)	**0.002**

Model 1 *, unadjusted; Model 2 ^†^, adjusted for age, sex; Model 3 ^§^, adjusted for age, sex, alcohol drinking, exercise, body mass index, diabetes, hypertension, dyslipidemia, and presence of cirrhosis. Abbreviations: MAFLD, metabolic-associated fatty liver disease; HR, hazard ratio; HCC, hepatocellular carcinoma; CVD, cardiovascular disease.

## Data Availability

The datasets generated during and/or analyzed during the current study are available from the corresponding author on reasonable request.
